# Interdisziplinäre Neukonzeption der Lehre im Querschnittsfach „Allergologie“ im Studiengang Humanmedizin

**DOI:** 10.1007/s00106-022-01222-5

**Published:** 2022-10-04

**Authors:** Marie Charlotte Schuppe, Christiane Lex, Nina Gliem, Ulrike Olgemüller, Michael Koziolek, Susann Forkel, Sidhi Gupta, Tobias Dombrowski, Bozena A. Czech-Zechmeister, Johannes Geier, Caroline Beutner, Timo Buhl

**Affiliations:** 1 Klinik für Dermatologie, Venerologie und Allergologie, Universitätsmedizin Göttingen, Robert Koch Str. 40, 37075 Göttingen, Deutschland; 2 Klinik für pädiatrische Kardiologie, Intensivmedizin und Neonatologie, Universitätsmedizin Göttingen, Göttingen, Deutschland; 3 Klinik für Gastroenterologie, gastrointestinale Onkologie und Endokrinologie, Universitätsmedizin Göttingen, Göttingen, Deutschland; 4 Klinik für Kardiologie und Pneumologie, Universitätsmedizin Göttingen, Göttingen, Deutschland; 5 Klinik für Nephrologie und Rheumatologie, Universitätsmedizin Göttingen, Göttingen, Deutschland; 6 Klinik für Hals-Nasen-Ohrenheilkunde, Universitätsmedizin Göttingen, Göttingen, Deutschland; 7 Institut für Klinische Chemie, Universitätsmedizin Göttingen, Göttingen, Deutschland; 8 Informationsverbund Dermatologischer Kliniken (IVDK), Universitätsmedizin Göttingen, Göttingen, Deutschland; 9 Niedersächsisches Institut für Berufsdermatologie (NIB), Universitätsmedizin Göttingen, Göttingen, Deutschland

**Keywords:** Allergologie, Lehre, Humanmedizin, Comprehensive Allergy Center, Studierende, Allergology, Education, Medical study, Comprehensive Allergy Center, Students

## Abstract

**Hintergrund:**

Obwohl allergologische Erkrankungen zu den wichtigen Gesundheitsstörungen laut ärztlicher Approbationsordnung zählen, ist die Allergologie in Deutschland nicht als selbstständiges Fach im Studium der Humanmedizin verankert.

**Ziel der Arbeit:**

Da sämtliche Universitäts- und Hochschulstandorte mit dieser Herausforderung umgehen müssen, war es Ziel unseres Lehrprojekts, eine exemplarische und mit allen beteiligten Kliniken und Instituten abgestimmte Koordination und Verzahnung der allergologischen Lehre an einem Standort zu etablieren. Insbesondere Comprehensive Allergy Centers (CAC) bieten eine bereits vorhandene Infrastruktur, in der diese Neukonzeption der allergologischen Lehre auf andere Standorte übertragen werden könnte.

**Material und Methoden:**

Nach umfangreicher Bestandsaufnahme der aktuellen allergologischen Lehre an der Universitätsmedizin Göttingen wurde im interdisziplinären Konsens ein neues Lehrkonzept entwickelt, durch die Bereitstellung zusätzlicher digitaler Lehr- und Lernanteile ergänzt („blended learning“) und schließlich evaluiert.

**Ergebnisse:**

Die allergologische Lehre im klinischen Studienabschnitt zeigte eine starke Fragmentierung, die ohne Koordination der zwölf beteiligten Kliniken/Institute und ohne Abstimmung der jeweiligen Lerninhalte stattfand. In der etablierten Struktur des interdisziplinären CAC erfolgte eine Neukonzeption, Koordination und Schwerpunktsetzung der studentischen Lehre zur klinischen Allergologie. Die Bereitstellung von neuen interaktiven Lerneinheiten sowie ergänzender Materialien zum Selbststudium wurden von den Studierenden positiv bewertet. Eine vergleichende Evaluation von Studierenden nach Absolvieren der unterschiedlichen Curricula zeigte signifikante Verbesserungen im Erreichen der gewünschten Lernziele.

## Abstract (en)

**Background:**

Although allergic diseases are among the most important health disorders, allergology is not anchored as an independent subject in the clinical part of medical studies in Germany.

**Objective:**

As all universities and institutes face the same challenge, the aim of our project was to establish exemplary coordination and networking of education in allergology at one location in agreement with all involved departments and institutes. Particularly, Comprehensive Allergy Centers (CAC) offer an established infrastructure via which the revised allergology education program can be transferred to other universities.

**Materials and methods:**

After an extensive inventory of the current allergological curriculum at the University Medical Center Göttingen, a new teaching concept was developed in interdisciplinary consensus, supplemented by first-time provision of additional digital contents (“blended learning”), and finally evaluated.

**Results:**

Initially, we observed a high level of fragmentation in the teaching of allergology in the clinical study sections of human medicine, with no coordination between the 12 clinical departments/institutes involved and no coherent framework for the specific learning content. Within the established structure of the interdisciplinary CAC, we revised, coordinated, and defined key areas for improved student education in clinical allergology. The allocation of new interactive learning elements as well as supplementary materials for self-studies was welcomed by the students and positively evaluated. A survey among students after completing the former vs. current curricula showed significant improvements in achieving the desired educational objectives.

## Hintergrund und Fragestellung

Allergische Erkrankungen stellen eine zunehmende gesundheitliche Problematik unserer modernen Gesellschaft dar [1]. Eine steigende Prävalenz von allergischen Sensibilisierungen [2, 4] sowie neue Erkenntnisse über Erkrankungen aus dem allergologischen Formenkreis sind Grundlage für einen wachsenden Bedarf an Wissen über die gesamte Bandbreite der verschiedenen medizinischen Fachgebiete. Dieser Bedarf wird auch in der medizinischen Ausbildung formuliert, denn in der ärztlichen Approbationsordnung werden allergische Erkrankungen explizit unter den wichtigsten Krankheitsbildern und Gesundheitsstörungen aufgeführt, die in der medizinischen Lehre abgebildet werden sollen [6]. Anders als in vielen europäischen Ländern ist das Fach Allergologie in Deutschland kein eigenständiges Gebiet der Facharztweiterbildung. Die Zusatz-Weiterbildung „Allergologie“ ist in ihrer Ausbildung schwerpunktmäßig der jeweiligen Facharztrichtung zugeordnet [8, 11]. Ebenso findet die allergologische Lehre an den Hochschulen in aller Regel nicht in einem eigenen Modul, sondern in verschiedenen Fachbereichen über den gesamten klinischen Studienabschnitt statt. Eine Koordination von Lerninhalten ist durch die fehlende Verankerung in einem eigenen Modul deutlich erschwert.

Im Rahmen eines mit Landesmitteln geförderten Projekts zur Verbesserung der studentischen Lehre in einem Comprehensive Allergy Center (CAC) erfolgte eine Revision der Lehre der klinischen Allergologie an der Universitätsmedizin Göttingen (UMG). Zielsetzung waren neben der Verbesserung der Struktur der Lehre insbesondere die longitudinale Vernetzung der über viele Semester verteilten Lehreinheiten. Digitalisierte klinische Fälle mit integrierten Lerninhalten und Übungseinheiten sollen bestehende bereits bewährte Lehrmethoden im Rahmen des neuen Lehrkonzepts unterstützen. Die am Standort Göttingen konzipierten Lehrinhalte erlauben eine Übertragung auch auf andere Hochschulstandorte.

## Methoden

Initial erfolgte eine detaillierte Bestandsaufnahme der historisch gewachsenen allergologischen Lehre in den verschiedenen Disziplinen der UMG. Orientiert am Veranstaltungskatalog für den klinischen Studienabschnitt im Fach Humanmedizin im Wintersemester 2020/2021 in Annahme einer regelhaften Präsenzlehre erfolgte eine Abfrage der Lehrveranstaltungsstunden der allergologisch Dozierenden unter Einschluss von Seminaren und Vorlesungen [16, 17]. Inhalte aus Kursen zu „Untersuchungen am Krankenbett“ (UaK), Wahlpflichtfächern und dem Praktischen Jahr wurden ausgeschlossen.

Zur studentischen Evaluation der bisherigen allergologischen Lehre erfolgte eine Befragung von 37 Studierenden zum Ende des klinischen Studienabschnitts mittels eines standardisierten Online-Fragebogens.

Die Erarbeitung eines neuen interdisziplinären Lehrkonzepts erfolgte in Zusammenarbeit der beteiligten Kliniken und Institute sowie der jeweils verantwortlichen DozentInnen. Für die Auswahl der Themeninhalte und Schwerpunkte wurden orientierend der Nationale Kompetenzbasierte Lernzielkatalog (NKLM, 2015) [10], der Gegenstandskatalog für die zweite ärztliche Prüfung des IMPP (IMPP-GK 2, Auflage 4, 2003) [12] sowie der lokale Lernzielkatalog für den klinischen Studienabschnitt der UMG [16, 17] genutzt. Zudem wurde der neue Masterplan Medizinstudium 2020 zu vorgesehenen Veränderungen der ärztlichen Ausbildung zu allergologischen Themeninhalten einbezogen [5]. Zentraler Bestandteil des neu erarbeiteten Lehrkonzepts wurde darüber hinaus die Bereitstellung von digitalen Lerninhalten, begleitend zu den Lehrveranstaltungen im interdisziplinären Curriculum. Auf der universitätsweit eingesetzten web-/appbasierten Lernplattform ILIAS (Open Source Software, Version 5.3.17, https://www.ilias.de/) wurde im Sommersemester 2021 erstmalig ein verändertes und erweitertes Lernangebot mit interaktiven Fallbeispielen, zusätzlichen Materialien zum Selbststudium sowie Lernkontrollen zur Verfügung gestellt.

Nach Erstellung des neuen interdisziplinären Lehrkonzepts erfolgte der Vergleich des aktuellen Stands der allergologischen Lehre mit der Zielvorstellung, welcher sowohl Überschneidungen als auch inhaltliche Defizite des gesamten, allergologisch abzudeckenden Spektrums ergab. Die somit sinnvollen und notwendigen Umstrukturierungen sowie neue Inhalte und Schwerpunkte wurden mit allen beteiligten Kliniken und Instituten konstruktiv diskutiert sowie eine Umsetzung des Lehrkonzepts in der zur Verfügung stehenden Lehrzeit geplant. Darüber hinaus wurden den jeweiligen DozentInnen Muster für die Erstellung weiterer digitaler Lerninhalte bereitgestellt. Da der größte Anteil der Lehrzeit in der Allergologie in der UMG auf das 4. klinische Semester entfällt, erfolgte hier die erste Anpassung der Veranstaltungsformate. Studierende erhielten erstmalig Zugriff auf zusätzliche digitale Lernformate. Neben der Erfassung der Wahrnehmung der bisherigen Lehre zum Ende des klinischen Studienabschnitts wurde nach Etablierung des neuen Lehrkonzepts im 4. klinischen Semester eine Evaluation durch 59 andere Studierende wiederum mittels eines standardisierten Online-Fragebogens durchgeführt. Hierbei wurden neben Fragen zur Selbsteinschätzung von Kenntnisstand und Lernzuwachs auch Aspekte der Organisation und Didaktik sowie die Bereitstellung digitaler Lerninhalte mittels Likert-Skala erfasst. Eine vergleichende inhaltliche Lernzuwachskontrolle zur Objektivierung des Wissenszuwachses war nicht Teil der studentischen Evaluation. Enthaltungen wurden in den Auswertungen nicht berücksichtigt. Die Befragung der Studierenden wurde von der Ethikkommission der UMG genehmigt (Antragsnummer 31/5/21). Die statistische Auswertung erfolgte mit dem Software-Programm R (Version 1.2.5033, https://www.r-project.org/, RRID:SCR_001905). Gruppenunterschiede wurden mittels Wilcoxon-Vorzeichen-Rang-Test auf Signifikanz getestet. Eine direkte Bewertung der digitalen Lernformate auf der Lernplattform ILIAS wurde durch Bewertung einzelner Lernabschnitte sowie durch Nutzung der Kommentarfunktionen ermöglicht.

## Ergebnisse

**Figure 1:**
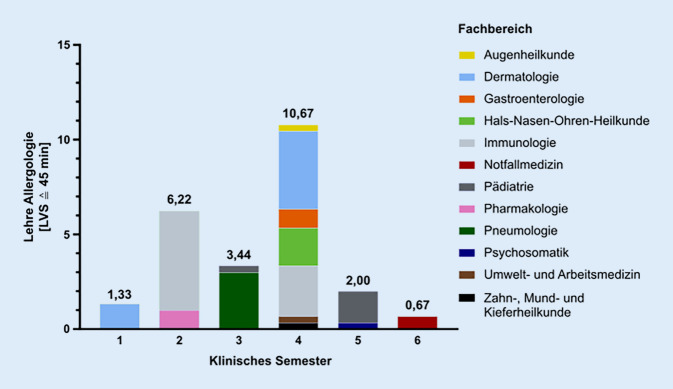

Die traditionell gewachsene Struktur der Allergologie als Querschnittsfach ist mit einer starken Fragmentierung der Lehre verbunden. Laut Veranstaltungskatalog für das Fach Humanmedizin an der UMG in Annahme einer regelhaften Präsenzlehre im Wintersemester 2020/2021 erfolgt die Lehre der Allergologie durch zwölf verschiedene Kliniken/Institute verteilt auf zehn klinische Module über den gesamten klinischen Studienabschnitt. Besonders viele Abteilungen lehren allergologische Inhalte im 4. klinischen Semester, wobei diese zeitliche Zuordnung der Fachlehre übergeordnet vorgegeben ist und in diesem Projekt nicht verändert werden konnte (Abb. 1). Anteile der allergologischen Lehrveranstaltungen werden neben den CAC-Kliniken durch weitere Institutionen oder externe DozentInnen übernommen. Am Standort Göttingen ist beispielsweise der Informationsverbund Dermatologischer Kliniken (IVDK) als An-Institut an der Vermittlung von chronisch-entzündlichen Hautkrankheiten und Berufserkrankungen beteiligt. Insgesamt erfolgt die allergologische Lehre über den gesamten klinischen Studienabschnitt in 24 Lehrveranstaltungsstunden (LVS) mit einer Dauer von jeweils 45 min. Eine Koordination der einzelnen Fachbereiche oder eine Abstimmung der spezifischen Lerninhalte und Themenschwerpunkte erfolgte an der UMG bisher nicht.

**Figure 2:**
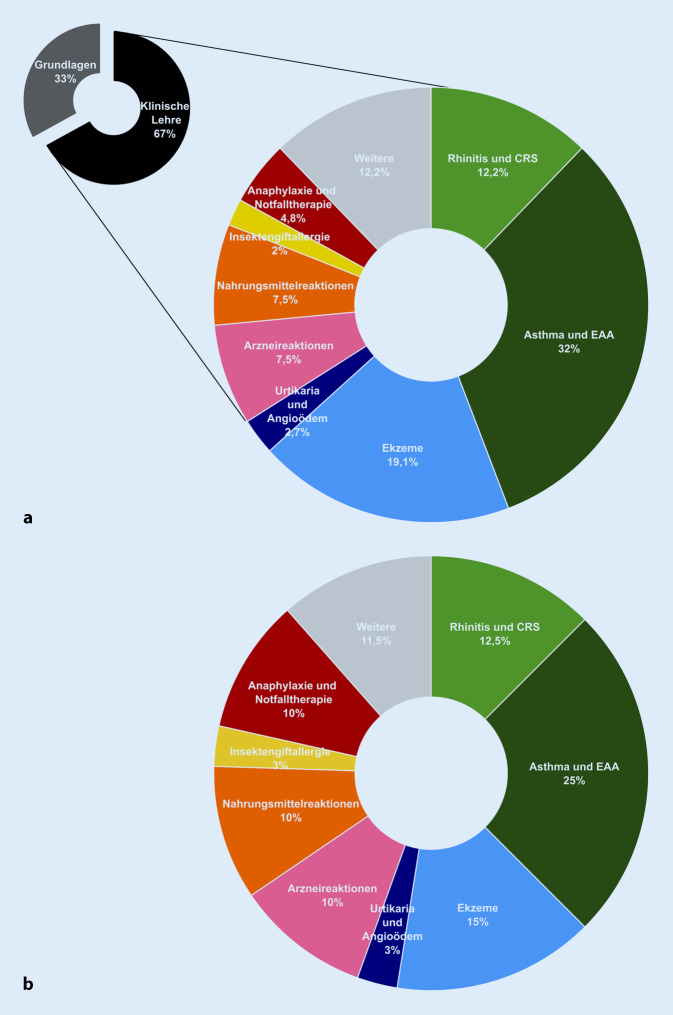

Die Erfassung des Ist-Zustands der Inhalte ergab, unabhängig von der lehrenden Abteilung, dass Krankheitsbilder wie Asthma bronchiale oder allergische Kontaktekzeme ausführlich behandelt wurden (Abb. 2a). Andere im klinischen Alltag relevante Themen wie Nahrungsmittelallergien oder Arzneimittelreaktionen waren in geringerem Umfang in der klinischen Lehre vertreten. In der Bestandsanalyse der Lehrenden aller CAC-Kliniken zeigte sich ebenfalls, dass im Themenfeld „Anaphylaxie und Notfalltherapie“ auch nach Absolvieren der klinischen Lehreinheiten deutliche Lücken im Kenntnistand der Studierenden bestanden und somit eine zu geringe Gewichtung in der allergologischen Lehre offensichtlich wurde. Die Notwendigkeit einer Revision des bisherigen Lehrkonzepts wurde auch durch eine Befragung von Studierenden kurz vor dem Praktischen Jahr bestätigt. Nur 10,8 % (4/37) der befragten Studierenden sahen die bisherige allergologische Lehre im klinischen Studienabschnitt gut abgebildet. Auch bei digitalen Lerninhalten wurde ein Verbesserungsbedarf deutlich, denn nur 16,2 % (6/37) der Studierenden fanden, dass die Präsenzlehre gut durch zusätzlich digitale Angebote ergänzt wurde.

Zur Entwicklung eines strukturierten, neuen interdisziplinären Lehrkonzepts mit Möglichkeit der Anpassung an sich stetig verändernde Curricula wurden die beteiligten Fachbereiche durch das CAC an der UMG koordiniert. Zunächst wurde das Ergebnis der Bestandsaufnahme der Lehre in der Allergologie im Hinblick auf die zur Verfügung stehende Lehrzeit sowie redundante und bisher nicht abgebildete Themeninhalte diskutiert. Mit etwa acht Lehreinheiten je 45 min (entsprechend 33 % der gesamten Lehrzeit) hat das theoretische „Institut für zelluläre und molekulare Immunologie“ den größten Anteil an der allergologisch-immunologischen Lehre. In zwei verschiedenen klinischen Modulen findet in diesen Lehreinheiten die Vermittlung grundlegender Kenntnisse der komplexen immunologischen Zusammenhänge allergischer Erkrankungen statt, welche nach Einschätzung aller beteiligter Fachbereiche als essenziell für die ärztliche Ausbildung zu werten sind und damit unverändert bleiben. Daher lag in diesem Projekt der Fokus auf den klinischen Lehrinhalten. Ebenso einigten sich alle beteiligten Fachbereiche, dass an der zur Verfügung stehenden absoluten allergologischen Lehrzeit je Klinik/Institut keine Veränderungen vorgeschlagen werden sollten.

**Figure 3:**
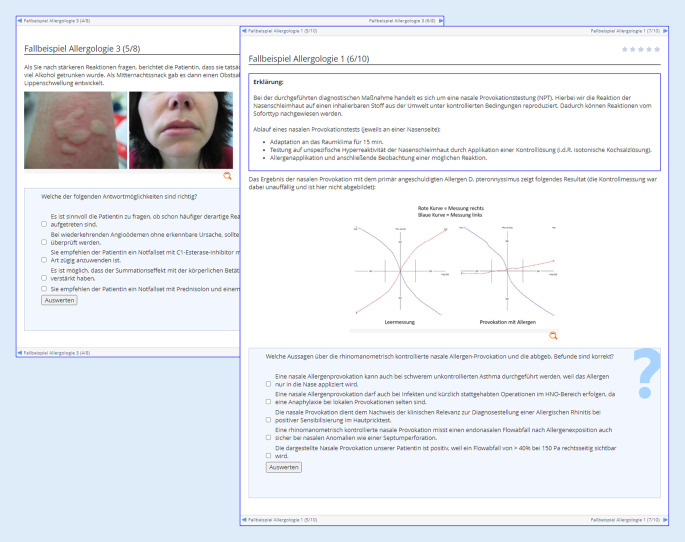

**Figure 4:**
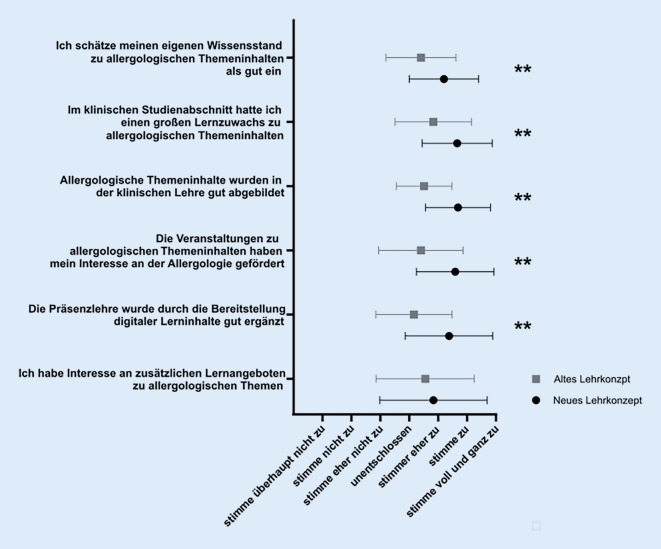

Über alle Kliniken und Institute mit allergologischen Lehrinhalten verblieben somit 16 Lehreinheiten (entsprechend 67 % der gesamten Lehrzeit Allergologie), welche einer Umstrukturierung unterzogen wurden. Nach Zusammenfassung der Lehrinhalte in größere Themenkomplexe wurde eine neue Gewichtung gemeinsam im CAC festgelegt. Neben den Vorgaben aus den verschiedenen Curricula kam hier die Heraushebung besonders wichtiger Schlüsselkompetenzen zum Tragen. Weiterhin stellen allergische Erkrankungen der oberen und unteren Atemwege sowie Ekzemerkrankungen Hauptthemenschwerpunkte dar, wurden jedoch hinsichtlich des Lehranteils zugunsten unterrepräsentierter Themenfelder deutlich angepasst. Diese konsentierten prozentualen Lehrzeiten beinhalteten bewusst eine redundante Abhandlung zentraler Lerninhalte innerhalb unterschiedlicher Veranstaltungsformate für eine bessere interdisziplinäre Vernetzung (Abb. 2b). Zusätzlich wurden für die Vertiefung des gelernten Wissens neue digitale Lernformate erarbeitet, in denen neben interaktiven Fallbeispielen mit Fragen zur Lernstandkontrolle weitere detaillierte Erklärungen sowie Materialien zum Selbststudium zur Verfügung gestellt werden. Die Bearbeitung der digitalen Lernformate soll das Wissen aus den Präsenz-Veranstaltungen in den klinischen Modulen rekapitulieren und die Kompetenzen der Studierenden für allergologische Themen festigen. Zudem werden in diesen Lernformaten alltägliche klinische Fragestellungen und Befundkonstellationen als Vorbereitung auf die spätere ärztliche Tätigkeit simuliert (Abb. 3). Für das Sommersemester 2021 wurde das neu erarbeitete interdisziplinäre Lehrkonzept im 4. klinischen Semester etabliert. Hierfür wurden die Lehrinhalte entsprechend den festgelegten Themenschwerpunkten und Schlüsselkompetenzen angepasst. Zudem erhielten die Studierenden erstmalig Zugang zu den neuen digitalen Lernformaten im „Lern Content Management System“ ILIAS, das als grundsätzliche Lernplattform an der UMG über alle Disziplinen genutzt wird. Die zur Evaluation durchgeführte Umfrage unter den Studierenden ergab eine positivere Bewertung des neuen Curriculums. Anders als bei vielen Lehr-Evaluationsstudien wurde hier nicht der Lernzuwachs einer Studierenden/eines Studierenden vor und nach Absolvieren einer Lehreinheit longitudinal untersucht. Stattdessen wurde eine Studierendengruppe nach Absolvieren des alten Curriculums (*n* = 37) mit anderen Studierenden nach Absolvieren des neuen Allergologie-Curriculums (*n* = 59) verglichen (Abb. 4). Während im alten Curriculum nur 34,1 % der Studierenden einen großen Lernzuwachs in der Allergologie beschrieben, gaben im neuen Curriculum 62,7 % der Befragten einen großen Lernzuwachs an. Noch deutlicher zeigten sich die Verbesserungen bei der Frage nach der „Abbildung allergologischer Themeninhalte im klinischen Studienabschnitt“: Dies bewerteten 10,8 % der Studierenden im alten Curriculum und 66,1 % der Befragten im neuen Curriculum mit „gut“. Im Vergleich zur Einschätzung der Studierenden aus dem 5. und 6. klinischen Semester konnte durch die Neukonzeption demnach bereits ein signifikant größerer Lernzuwachs zu allergologischen Themeninhalten und eine verbesserte Repräsentation der Allergologie in der klinischen Lehre erreicht werden. Mehr als die Hälfte (57,6 %) der befragten Studierenden sahen zudem die Präsenzlehre gut durch die neuen digitalen Lernformate ergänzt, sodass die Befragung auch hier eine signifikante Verbesserung durch das novellierte Lehrkonzept ergab.

## Diskussion

Während die Allergologie in vielen europäischen Ländern eine eigene Facharztausbildung für „Allergologie und klinische Immunologie“ mit klaren Vorgaben zur Aus- und Weiterbildung darstellt, existiert in Deutschland eine Zusatz-Weiterbildung „Allergologie“ im Anschluss an zahlreiche Facharztanerkennungen [7, 11]. Nicht nur in der ärztlichen Weiterbildung, sondern auch bereits in der studentischen Lehre hat die Allergologie somit einen komplexen Stand. In den Lernzielkatalogen und Curricula für den klinischen Abschnitt im Medizinstudium werden nur wenige allergologische Schlüsselkompetenzen formuliert und vermittelt, welche die Studierenden am Ende ihrer ärztlichen Ausbildung erlangt haben sollten [16, 17]. Somit fehlt in der Regel nicht nur eine zentrale Koordination der zahlreichen, an der Lehre beteiligten Fachbereiche, sondern auch eine interdisziplinäre Vernetzung und inhaltliche Abstimmung der einzelnen kurzen Lehrabschnitte. In der Wahrnehmung der Studierenden, aber auch der klinischen DozentInnen, wurde die Allergologie als interdisziplinäres Fach bisher nicht gut genug im klinischen Studienabschnitt abgebildet.

Mit der Gründung und Zertifizierung von CAC besteht bereits an einigen Hochschulstandorten eine institutionalisierte Zusammenarbeit der verschiedenen Kliniken und Institute bei der Versorgung von PatientInnen mit allergologischen Erkrankungen [15, 18]. Mit der Zunahme von individuellen Entscheidungen bei der Anwendung komplexer Systemtherapeutika („precision medicine“), aufgrund von gehäuft vorliegenden allergologischen/atopischen Erkrankungen über die Fächergrenzen hinweg („personalized medicine“), wird der gemeinsame Beratungs- und Entscheidungsbedarf zum therapeutischen Vorgehen in Zukunft erheblich zunehmen. Daher ist die bestehende Struktur eines CAC nicht nur für die Patientenversorgung und ärztliche Weiterbildung essenziell, sondern ebenso für eine strukturierte Vermittlung allergologischer Inhalte in der studentischen Lehre sehr gut geeignet. Jedoch könnten auch unabhängig von den strukturellen Gegebenheiten an den verschiedenen medizinischen Hochschulen in Deutschland Anteile unserer erarbeiteten Neukonzeption hierfür als Vorlage und Anregung dienen. Wir weisen darauf hin, dass wir den Umfang der allergologischen Lehrzeit im Gesamtlehreplan der einzelnen Kliniken nicht thematisiert haben. Somit geben wir in dieser Arbeit keine Stellung dazu ab, inwieweit der gesamte Lehrumfang der Allergologie an unserem Standort im Humanmedizinstudium passend ist.

Bereits kleinere und weniger zeitaufwendige Umstellungen der bisherigen allergologischen Lehre können zu einer deutlichen Sensibilisierung der Studierenden für Kenntnisse zu den wichtigen und komplexen allergischen Erkrankungen führen. Hierbei ist die Vernetzung von theoretischem Wissen und praktischen Fertigkeiten von zentraler Bedeutung. Die Nutzung fallbasierten Lernens zur Annäherung der Lehre an die klinische Tätigkeit hat sich als sehr gute didaktische Strategie zur Stärkung ärztlicher Kompetenzen erwiesen [9]. So stellt die Etablierung digitaler Lernformate mit interdisziplinären Fallbeispielen eine wertvolle Ergänzung bestehender Lehrmethoden dar [14]. Wir konnten in der Evaluation unserer erneuerten curricularen Lehre bereits eine signifikante Verbesserung in fünf von sechs Fragen in der Selbsteinschätzung zu allergologischen Themen durch die Studierenden erzielen. Der Aufwand hierfür ist deutlich kleiner einzuschätzen als grundsätzliche inhaltliche Überarbeitungen der Einzelfachlehre. Zusätzlich zur curricularen Lehre im klinischen Studienabschnitt können allergologische Lernangebote durch ein interdisziplinäres Wahlfach ergänzt werden [3].

Die Medizin befindet sich als multidisziplinäres Fach im Wandel der Zeit. Die ärztliche Ausbildung und somit auch die allergologische Lehre müssen somit an ständig neue Vorgaben angepasst werden. Der Masterplan Medizinstudium 2020 hat eine grundlegende Neustrukturierung des zukünftigen Medizinstudiums vorgesehen und den nationalen Lernzielkatalog (NKLM) als einheitlichen Zielrahmen hierfür festgelegt [5, 13, 19]. Noch ist unklar, ob und welche inhaltlichen Veränderungen für die Lehre allergologischer Themeninhalte vorgenommen werden und wann sie in den verschiedenen Curricula an den medizinischen Fakultäten in Deutschland zum Tragen kommen. Mit der koordinierten Revision der bisherigen Strukturen und Lehrinhalte konnten wir dem langfristigen Ziel einer besseren Verknüpfung von theoretischen Grundlagen und klinischer Medizin sowie einer guten fächerübergreifenden, kompetenzorientierten Lehre bereits jetzt näherkommen. Eine kontinuierliche und kritische Evaluation der Integration allergologischer Themeninhalte in die curriculare Lehre wird notwendig sein, um die Studierenden auf ihre spätere ärztliche Tätigkeit vorzubereiten und das Interesse an der Allergologie weiter zu fördern.

## Fazit für die Praxis

Durch neue Lehrformate und eine koordinierte Revision bisheriger Lerninhalte kann die allergologische Lehre mit vertretbarem Aufwand deutlich verbessert werden. Insbesondere CAC bieten eine bereits vorhandene Infrastruktur, in der unsere hier vorgestellte Neukonzeption der allergologischen Lehre auch als Anregung für andere Standorte genutzt werden kann.
